# Porous Fe_2_O_3_ Nanorods on Hierarchical Porous Biomass Carbon as Advanced Anode for High-Energy-Density Asymmetric Supercapacitors

**DOI:** 10.3389/fchem.2020.611852

**Published:** 2020-11-26

**Authors:** Pingping Yu, Wei Duan, Yanfeng Jiang

**Affiliations:** Department of Electronic Engineering, College of Internet-of-Things (IoT), Jiangnan University, Wuxi, China

**Keywords:** α-Fe_2_O_3_ (Hematite), biomass porous carbon, asymmetric supercapacitors (ASCs), aqueous electrolyte, high energy density

## Abstract

In this study, a novel negative electrode material was prepared by aligning α-Fe_2_O_3_ nanorods on a hierarchical porous carbon (HPC) skeleton. The skeleton was derived from wheat flour by a facile hydrothermal route to enhance conductivity, improve surface properties, and achieve substantially good electrochemical performances. The α-Fe_2_O_3_/HPC electrode exhibits enhanced specific capacitance of 706 F g^−1^, which is twice higher than that of α-Fe_2_O_3_. The advanced α-Fe_2_O_3_/HPC//PANI/HPC asymmetrical supercapacitor was built with an expanded voltage of 2.0 V in 1 M Li_2_SO_4_, possessing a specific capacitance of 212 F g^−1^ at 1 A g^−1^ and a maximum energy density of 117 Wh kg^−1^ at 1.0 kW kg^−1^, along with an excellent stability of 5.8% decay in capacitance after 5,000 cycles. This study affords a simple process to develop asymmetric supercapacitors, which exhibit high electrochemical performances and are applicable in next-generation energy storage devices, based on α-Fe_2_O_3_ hybrid materials.

## Introduction

Supercapacitors are efficient energy storage devices, and are clean and renewable with high efficiency, fast charge/discharge capability, and good cycling stability, which meet the increasing demands in various portable electronic devices (Zhai et al., 2011; Yu et al., 2015; Chen et al., 2020). Supercapacitors might be more practical when they obtain high-energy density at the same time to retain high specific power. It can increase the work voltage or the specific capacitance to enhance the energy density (Ike et al., 2015; Liu et al., 2016). Organic electrolytes or non-aqueous electrolytes with wider working voltages (3–4 V) suffer from lower ion conductivity and solvated ion size, which limits their future application (Meng et al., 2019; Zhang et al., 2019). Therefore, considerable efforts have to focus on fabrication of asymmetric supercapacitors (ASCs) based on the desirable negative and positive electrode with good electrochemical characteristics.

Various positive electrode materials have been used in ASC devices, such as carbon materials, transition metal oxides, and especially conducting polymers, with facile synthesis, low cost, and environmentally benign properties (Benzigar et al., 2018; Gao et al., 2019; Luo et al., 2019; Hong et al., 2020). In addition, multiscale porous carbon composites with conducting polymers have emerged as attractive electrodes in ASC devices owing to their high conductivity, abundant redox reactions, and mechanical stability (Yu M. H. et al., 2016; Meng et al., 2017; Jin et al., 2018). Recently, polyaniline nanorods directly grown on porous carbon derived from wheat flours demonstrated excellent supercapacitive performance (Wu et al., 2015; Yu P. P. et al., 2016; Yu et al., 2019), and thus may serve as a good cathode for ASCs.

Compared with the extraordinary advancement of anode nanomaterials that rely on high activity and large potential ranges, the lack of highly performing cathodes is still a bottleneck for making progress in advanced ASCs. Carbon materials play a role as negative electrodes, using the electrical double layer to accumulate energy, while the specific capacitance is trapped at an intermediate level of 100–200 F g^−1^ (Zhu et al., 2011; Sahu et al., 2017; Wang et al., 2019). Recent studies have reported that pseudocapacitive anodes can deliver higher charge storage capacities than carbon materials, such as MoO_3_ (Zhang et al., 2019), V_2_O_5_ (Guo et al., 2015), WO_3_ (Yun T. G. et al., 2019), TiN Sun et al., 2020, Fe_3_O_4_ (Arun et al., 2019), FeOOH (Chen et al., 2016), and Fe_2_O_3_ (Yun X. et al., 2019; Le et al., 2020; Zhang et al., 2020). Among them, α-Fe_2_O_3_ is chosen as a potential candidate for ASCs because of its natural abundance, eco-friendly nature, excellent physicochemical stability, large theoretical capacitance (~3,625 F g^−1^), and high hydrogen evolution potential in aqueous solution (Han et al., 2014; Nithya and Arul, 2016; Li et al., 2019). Its pseudocapacitive properties arise from the redox behaviors of the Fe^3+^/Fe^2+^ peaks (Han et al., 2018). However, agglomerating easily into large particles of α-Fe_2_O_3_ with low conductivity (10^−14^ S cm^−1^) increases the electron transfer resistance during repeated ion charge/discharge cycles, showing a large effect on supercapacitance (Chen et al., 2014). These drawbacks will result in a reduction in the rate capacity and lifespan due to volume expansion, impeding the practical application as a negative electrode. By constructing integrated hybrid electrodes, α-Fe_2_O_3_ is directly deposited onto conductive skeletons of carbonaceous materials. Carbon nanotubes (Dong et al., 2018; Yue et al., 2018), porous carbon (Arun et al., 2019), and graphene (Xu et al., 2019) can overcome this vexing issue of low conductivity, which indeed demonstrates that integrated hybrid electrodes show better electrochemical performances than individual components because of the synergistic effect between the α-Fe_2_O_3_ nanostructure and the carbon configurations. Thus, far, the unsatisfactory dispersion and small surface area of α-Fe_2_O_3_ still causes a relatively low specific capacitance. Therefore, superior α-Fe_2_O_3_/carbon composites with excellent electrochemical performance are desired as negative electrodes, posing a major challenge for the preparation and design of ASCs with good electrochemical properties.

**Figure 1:**
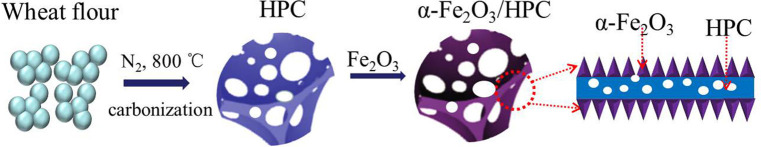
Schematic illustration of the fabrication of α-Fe_2_O_3_/hierarchical porous carbon (HPC) composites.

In this study, porous α-Fe_2_O_3_ nanowires were deposited onto conductive biomass hierarchical porous carbon (HPC) composites (Figure 1) by synthesizing via a hydrothermal process, and the nanowires acted as a Faradaic cathode with a pseudocapacitive anode (PANI/HPC) to assemble the ASC. HPC derived from wheat flour offers high conductivity and large surface area in both electrodes and thus improves the dispersion and stability of α-Fe_2_O_3_ and PANI nanowires, resulting in a good rate capacity. Porous α-Fe_2_O_3_ nanowires were well-arranged on the HPC surface directly, ensuring superior utilization and reduction in the ion diffusion length. The obtained α-Fe_2_O_3_/HPC//PANI/HPC ASC exhibited high energy/power density and electrochemical stability, indicating a wide prospect in future practical applications.

## Experiment Section

### Materials

All chemicals were used as achieved without treatment. Wheat flour was purchased from the Jingdong supermarket. KOH, Na_2_SO_4_, urea, Fe(NO_3_)_3_·9H_2_O, aniline, and ammonium persulfate were purchased from Sinopharm Chemical Reagent Co., Ltd.

### Synthesis of α-Fe_2_O_3_/HPC Composites

HPC was synthesized via a previously reported synthesis process (Yu P. P. et al., 2016). Briefly, the waste wheat flour (5 g), KOH (5 g), and urea (5 g) were mixed by stirring in 100 ml of distilled water (DI) before calcination in N_2_ flux at 800°C for 1 h. The obtained HPC was washed with 5% HCl and DI water and then dried at 80°C.

Typically, the yellow solution in a stainless-steel autoclave (100 ml) consisted of 1.75 mM Fe(NO_3_)_3_·9H_2_O, Na_2_SO_4_ (1.75 mM), and 50 g of deionized water. The mixture with uniformly dispersed HPC was heated in the autoclave to 120°C for 9 h. The obtained α-Fe_2_O_3_/HPC composites were added to DI water, collected by centrifugation, dried in vacuum at 60°C, and annealed under atmosphere at 300°C for 2 h. The α-Fe_2_O_3_ accounted for 35.4% of the weight of α-Fe_2_O_3_/HPC. The bare α-Fe_2_O_3_ was prepared via the same process without HPC.

### Synthesis of PANI/HPC Composites

Aniline monomers (0.07 M) and HPC (150 mg) were sequentially added to 1 M H_2_SO_4_ under strong stirring; then, the uniform ammonium persulfate (0.07 M) 1 M H_2_SO_4_ solution was quickly loaded in the above solution with stirring for 10 min. The mixed solution was placed at −2°C for 14 h. Finally, the resulting PANI/HPC composites were washed and dried at 60°C. The loaded PANI was 26.2% in PANI/HPC.

### Material Characterization

X-ray diffraction (XRD; Rifaku, Cu Kα radiation, 10–80°) and X-ray photoelectron spectroscopy (XPS; ESCA PHI 5000C) were used to analyze the crystalline and bonding energy of the samples. The morphology and structure were examined by scanning electron microscopy (SEM, Hitachi, SU8010) and transmission electron microscopy (TEM; JEOL JEM-2011). The Brunauer–Emmer–Teller (BET) specific surface area and pore size distribution of the composites were measured using a surface area analyzer (ASAP 2020, USA) and the Barrett–Joyner–Halenda (BJH) method.

### Electrochemical Measurements

A uniform paste was made by mixing HPC, α-Fe_2_O_3_/HPC, and PANI/HPC/carbon black/polytetrafluoroethylene at a ratio of 90/5/5, and was used to coat a 1.0-cm^2^ piece of titanium mesh as the working electrode. A three-electrode cell consisting of a saturated calomel electrode (SCE) and Pt foil was used to test the above sample paste. The ASCs were assembled by a negative electrode of α-Fe_2_O_3_/HPC and a positive electrode of PANI/HPC, and the separator was a Celgard 3501. The loaded mass of α-Fe_2_O_3_/HPC and PANI/HPC composite was about 2.1 and 2.5 mg, as per the charge balance theory. Electrochemical studies of all electrodes were performed using a CHI electrochemical workstation (660D) in 1 M Li_2_SO_4_ electrolyte. The specific capacitance (C_sp_) of HPC, α-Fe_2_O_3_/HPC, and PANI/HPC electrodes can be calculated depending on Equation (1):

(1) $$\begin{array}{r} C_{sp}=\frac{It}{mV} \end{array}$$

where Δt (s), I (A), ΔV (V), and m (g) stand for the discharge time, the current, the voltage difference, and the weight of the active materials.

The specific capacitance (C_asy_) of α-Fe_2_O_3_/HPC//PANI/HPC ASC is obtained from Equation (2),

(2) $$\begin{array}{r} C_{asy}=\frac{It}{MV} \end{array}$$

where M is the total mass of the electrode materials.

Energy density (E, Wh kg^−1^) and power density (P, W kg^−1^) of α-Fe_2_O_3_/HPC//PANI/HPC ASC are according to the equations:

(3) $$\begin{array}{r} E=\frac{C_{asy}V^{2}}{2\times 3.6} \end{array}$$

(4) $$\begin{array}{r} P=3600\times \frac{E}{t} \end{array}$$

## Results and Discussion

**Figure 2:**
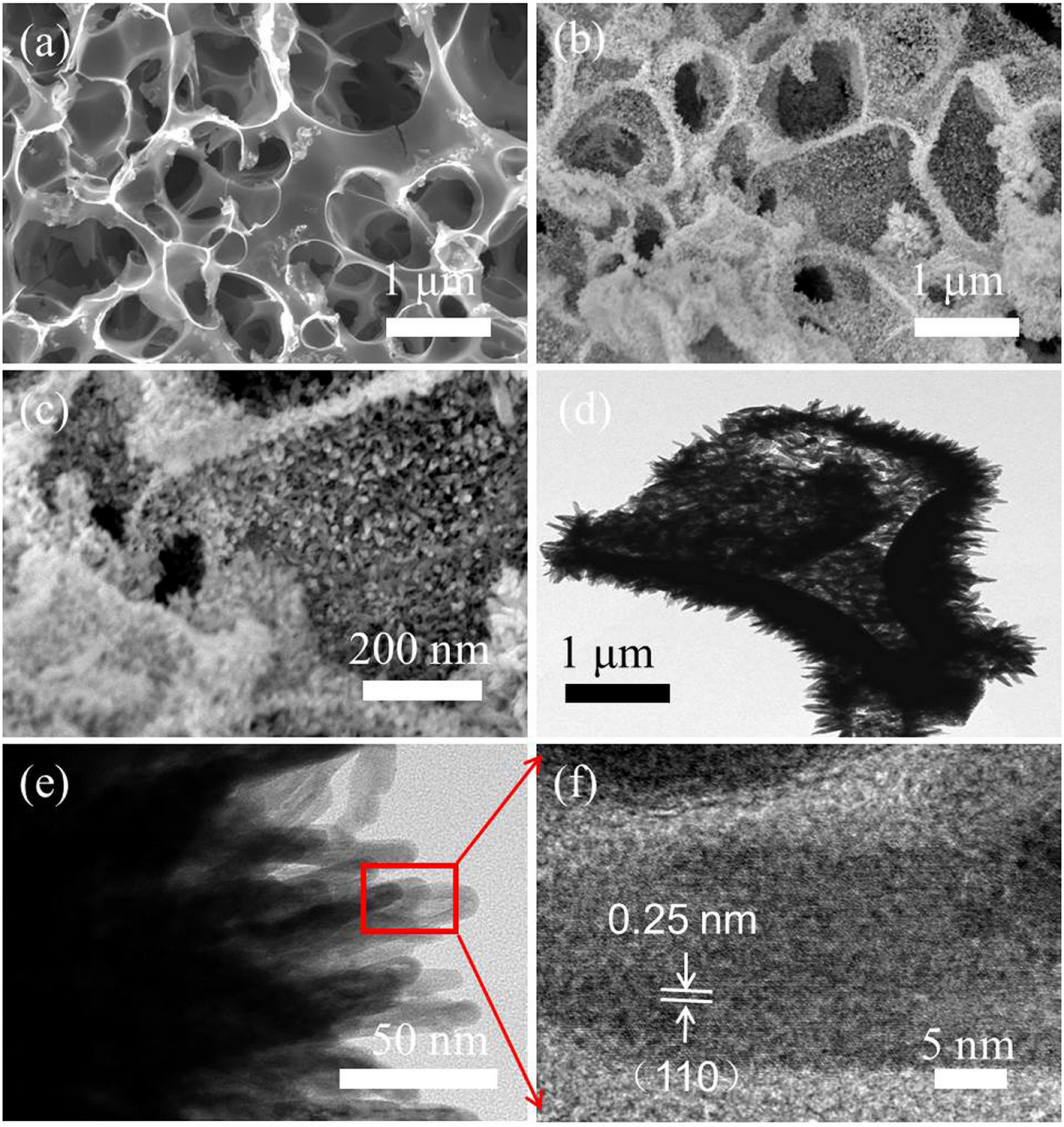
**(a)** Scanning electron microscopy (SEM) image of HPC, SEM images **(b,c)**, and transmission electron microscopy (TEM) images **(d,e)** of α-Fe_2_O_3_/HPC with the inset in **(e)** exhibiting the EDS spectrum. **(f)** High-resolution TEM (HRTEM) image of α-Fe_2_O_3_ from the red-marked region in **(e)**.

Figure 2a shows an interconnected hierarchical porous structure of HPC, similar to an open sponge with 100- to 200-nm thickness of carbon walls. The three-dimensional HPC served as a scaffold with high surface area and good conductivity for depositing α-Fe_2_O_3_ nanorods via a simple hydrothermal reaction. The α-Fe_2_O_3_ nanorods were directly and uniformly grown on the entire HPC surface (Figures 2b,c). The interior structure of α-Fe_2_O_3_/HPC was further tested via TEM (Figures 2d–f). The α-Fe_2_O_3_ nanorods, with diameters and lengths of ~10 and 50–100 nm, were vertically deposited on the scaffold of HPC to form a branched structure (Figure 2e), helping the fast transport of electrolyte ions. Moreover, the α-Fe_2_O_3_ nanorods have some pores (highlighted in the block diagram), resulting in a good specific surface to increase the number of accessible active sites for electrolyte ions. Meanwhile, high-resolution TEM (HRTEM) examination (Figure 2f) demonstrates the clear lattice fringes of the (110) plane of α-Fe_2_O_3_ with a spacing of 0.25 nm, indicating the high crystallinity of the α-Fe_2_O_3_ nanorods (Chen et al., 2015; Le et al., 2020).

**Figure 3:**
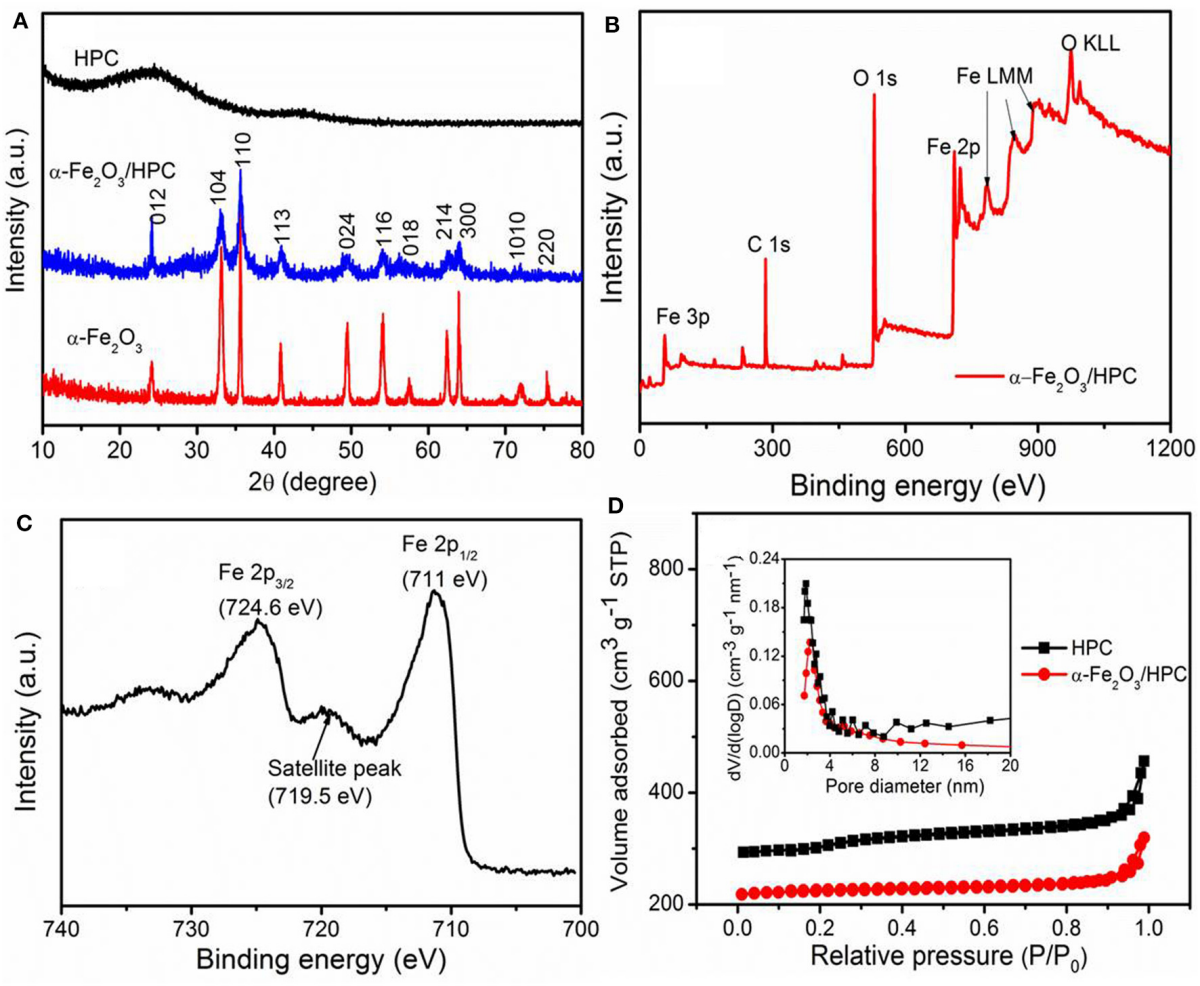
**(A)** X-ray diffraction (XRD) patterns of HPC, α-Fe_2_O_3_, and α-Fe_2_O_3_/HPC. **(B)** X-ray photoelectron spectroscopy (XPS) spectra of α-Fe_2_O_3_/HPC, **(C)** high-resolution Fe 2p XPS spectra. **(D)** The N_2_ adsorption–desorption (inset: pore size distributions) of HPC and α-Fe_2_O_3_/HPC.

Figure 3A shows XRD measurements to explore the structural and compositional properties of HPC, α-Fe_2_O_3_, and α-Fe_2_O_3_/HPC. The broad peaks of HPC at 2θ = 25° and 43.9° indicate the (002) and (101) planes of graphitized carbon (Yu P. P. et al., 2016; Liu et al., 2019). The other relevant characteristic diffraction peaks are very consistent with α-Fe_2_O_3_ (JCPDS 33-0664), which does not change in the α-Fe_2_O_3_/HPC composite. The detailed surface chemical composition of α-Fe_2_O_3_/HPC was evaluated by XPS measurements. Figure 3B demonstrates the coexistence of C (283.5 eV), O (527.6 eV), and Fe (Fe 2p peaks at 724.6 and 711 eV) (Yun X. et al., 2019; Racik et al., 2020). The high-resolution Fe 2p XPS spectrum exhibits typical Fe 2p_1/2_ and 2p_3/2_ peaks along with 719.5 eV of satellite peak corresponding to Fe^3+^ in α-Fe_2_O_3_ (Figure 3C) (Liang et al., 2018).

Figure 3D shows the N_2_ adsorption/desorption isothermal analysis of HPC and α-Fe_2_O_3_/HPC. The inset image shows the corresponding pore size distribution. The HPC and α-Fe_2_O_3_/HPC both exhibit IV type curves along with an H3 type hysteresis loop, which indicates the existing micropores and mesopores. Compared to HPC (977 m^2^ g^−1^), α-Fe_2_O_3_/HPC has a lower surface area of 700 m^2^ g^−1^, but is still superior to the reported carbon/α-Fe_2_O_3_ composites. The pore size distributions of α-Fe_2_O_3_/HPC were analyzed from the desorption isotherm, and a sharp peak was in the 1.0- to 4.0-nm range (inset of Figure 3D). This result is also confirmed by SEM and TEM images showing an interconnected multiple size porous structure to contribute to the large value of surface area, which ensures a good accessible electrolyte ion and facilitates ion transport.

**Figure 4:**
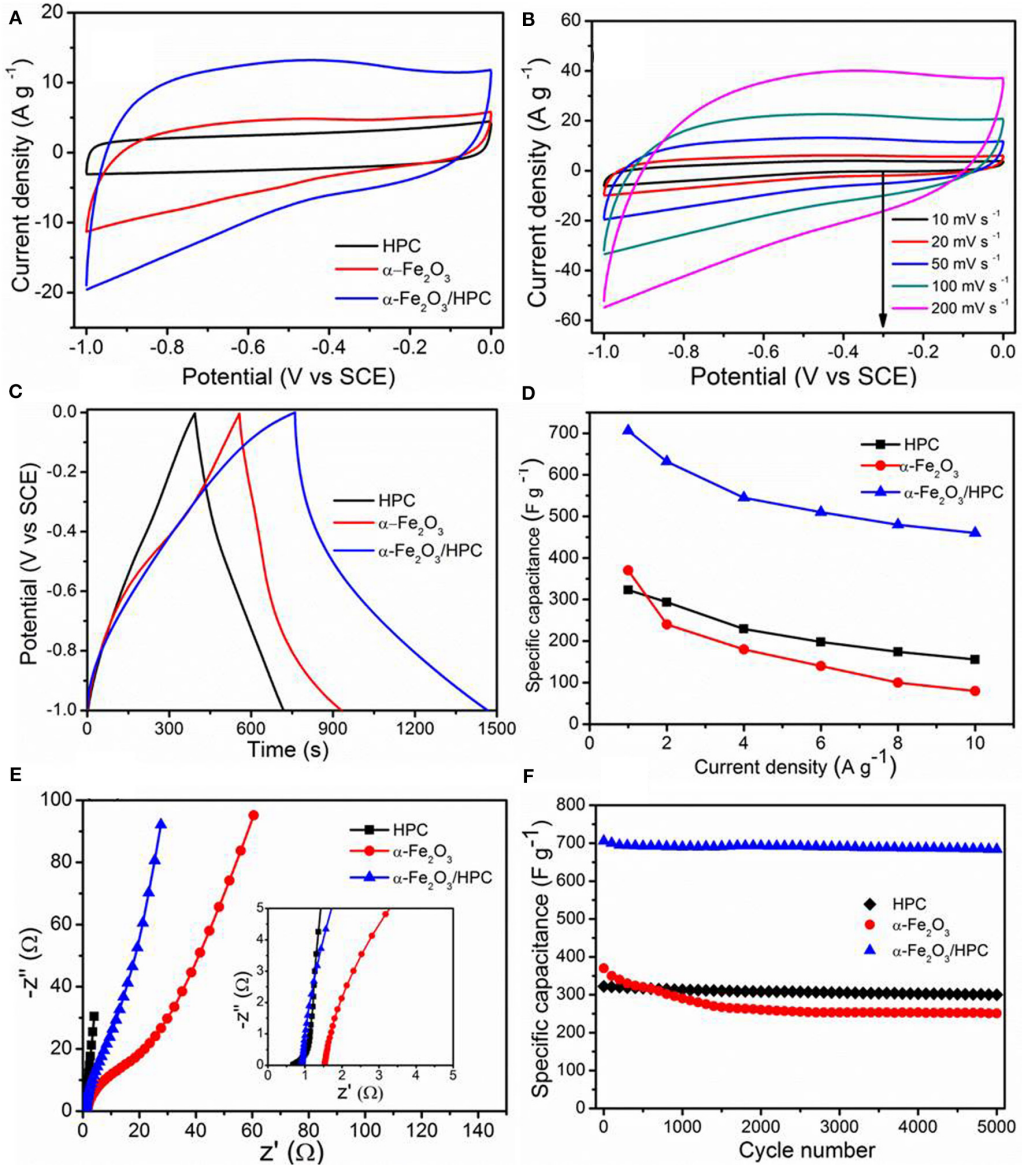
**(A)** Cyclic voltammetry (CV) curves of HPC, α-Fe_2_O_3_, and α-Fe_2_O_3_/HPC at 50 mV s^−1^. **(B)** CV curves of α-Fe_2_O_3_/HPC at different scan rates. **(C)** The galvanostatic charge/discharge (GCD) curves at a current of 1 A g^−1^, **(D)** gravimetric specific capacitance vs. current densities, **(E)** the Nyquist plots, and **(F)** cycling stability at 1 A g^−1^ of HPC, α-Fe_2_O_3_, and α-Fe_2_O_3_/HPC.

The electrochemical properties of HPC, α-Fe_2_O_3_, and α-Fe_2_O_3_/HPC electrodes were investigated through cyclic voltammetry (CV) curves, galvanostatic charge/discharge (GCD) plots, and electrochemical impedance spectroscopy (EIS) plots using 1 M Li_2_SO_4_ electrolyte in a three-electrode configuration. Figure 4A shows the compared CV curves of HPC, α-Fe_2_O_3_, and α-Fe_2_O_3_/HPC recorded at 50 mV s^−1^. A Nearly symmetrical rectangular shape demonstrates an ideal double-layer capacitor characteristic of the HPC electrode. Remarkably, the α-Fe_2_O_3_/HPC electrode shows a larger CV curve area than HPC and bare α-Fe_2_O_3_ nanorods, demonstrating a large enhancement in capacitive behavior owing to the synergistic effect on the double-layer capacitor characteristics of the HPC electrode and the pseudocapacitor of the α-Fe_2_O_3_ nanorods. There are no obvious anodic/cathodic peaks in the α-Fe_2_O_3_ and α-Fe_2_O_3_/HPC CV curves because of the pseudo-constant rate of charging/discharging throughout the entire voltammetric cycle, accompanied by a fast Faradic reaction between alkaline cations (Na^+^). This phenomenon results from the enhanced electrical conductivity and hierarchical porous structure resulting in fast ion diffusion under charge/discharge cycles. At 200 mV s^−1^, α-Fe_2_O_3_/HPC maintains a shape similar to that of the CV curves, demonstrating good capacitive performance (Figure 4B). Nearly symmetrical GCD profiles at the current density range of 1–10 A g^−1^ indicate the high coulombic efficiency (Supplementary Figure 1), which is consistent with CV curves.

Figure 4C shows the GCD plots of HPC, α-Fe_2_O_3_, and α-Fe_2_O_3_/HPC electrodes collected at 1 A g^−1^. Longer discharge time of the α-Fe_2_O_3_/HPC electrode indicates a higher capacitance than the HPC and α-Fe_2_O_3_ electrodes, convincingly revealing enhanced conductivity from HPC for the disposition of active α-Fe_2_O_3_ nanorods, which is consistent with the above CV curve analysis. This phenomenon demonstrates that the skeleton of HPC can improve the capacitance performance of α-Fe_2_O_3_, reduce the internal resistance between the interfacial and HPC, and offer more active sites for pseudocapacitors. Figure 4D shows the calculated C_sp_ values of HPC, α-Fe_2_O_3_, and α-Fe_2_O_3_/HPC electrodes under different current densities. Compared to the values of bare Fe_2_O_3_ nanorods (370 A g^−1^) and HPC (323 A g^−1^) electrodes at 1 A g^−1^, the maximum C_sp_ of the α-Fe_2_O_3_/HPC hybrid electrode is 706 F g^−1^, indicating a larger capacitive property. This result is superior to other recently reported iron oxide-based electrodes at the same current density, such as Fe_2_O_3_/graphene (226 F g^−1^) (Wang et al., 2013), Fe_2_O_3_/CNT (204 F g^−1^) (Yue et al., 2018), Fe_2_O_3_/N-doped CNT (264 F g^−1^) (Gnana Sundara Raj et al., 2020), α-Fe_2_O_3_/C (280 F g^−1^) (Dong et al., 2018), and Fe_2_O_3_/hemp straw (256 F g^−1^) (Jiang et al., 2020). At 10 A g^−1^, the C_sp_ of α-Fe_2_O_3_/HPC is 410 F g^−1^, showing a decrease as the current density increases. This value retains 58% of the initial capacitance, exhibiting a high-rate capability of α-Fe_2_O_3_/HPC, which is comparable to other reported electrodes such as Fe_2_O_3_/graphene (40% at 10 A g^−1^) (Wang et al., 2013), Fe_2_O_3_ nanospheres/diatomite (42% at 10 A g^−1^) (Jiang et al., 2018), and SiC@ Fe_2_O_3_ (51% at 12 A g^−1^) (Zhao et al., 2018). EIS analyses of HPC, α-Fe_2_O_3_, and α-Fe_2_O_3_/HPC electrodes were conducted. The charge transfer resistance of α-Fe_2_O_3_/HPC (0.91 Ω) was much smaller than that of bare α-Fe_2_O_3_ (1.63 Ω) and higher than that of pure HPC (0.52 Ω), indicating the enhanced conductivity of α-Fe_2_O_3_/HPC after adding the high conductive HPC as the scaffold (Figure 4E). The α-Fe_2_O_3_/HPC electrode has a more ideal straight line with small Warburg resistance, leading to fast transfer of electrolyte ions into hybrid electrode. Figure 4F shows the corresponding cycling stability of HPC, α-Fe_2_O_3_, and α-Fe_2_O_3_/HPC, evaluated at 1 A g^−1^. Notably, the cycling stability of the α-Fe_2_O_3_/HPC electrode is 95.8% of its original capacitance over 5,000 cycles, which is higher than 68% of the bare α-Fe_2_O_3_ electrode.

**Figure 5:**
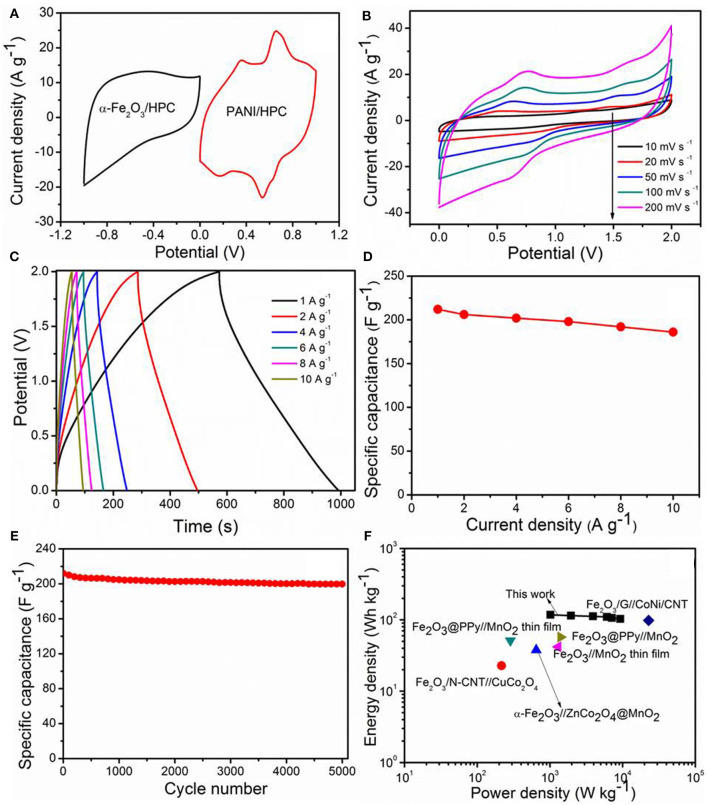
**(A)** CV curves of α-Fe_2_O_3_/HPC and pseudocapacitive anode (PANI)/HPC at 50 mV s^−1^. Electrochemical properties of α-Fe_2_O_3_/HPC//PANI/HPC asymmetric supercapacitors (ASC). **(B)** CV curves at various scan rates, **(C)** GCD curves, and **(D)** capacitance curves against current densities, **(E)** cycling stability at 1 A g^−1^, and **(F)** Ragone plots.

To further evaluate the possibility of α-Fe_2_O_3_/HPC electrode materials in practical applications of energy storage, the ASC device was built with α-Fe_2_O_3_/HPC and PANI/HPC as the cathode and anode, respectively, to achieve a high voltage range in 1 M Li_2_SO_4_ electrolyte. PANI nanorod arrays were aligned on interconnected porous surfaces of HPC through *in situ* polymerization (Supplementary Figures 2A,B), which shows a C_sp_ of 506 F g^−1^ at 10 mV s^−1^ measured in 1 M Li_2_SO_4_ electrolyte (Supplementary Figure 2C). The C_sp_ of PANI/HPC dropped to 343 F g^−1^, presenting capacitance retention of 67.8% at 200 mV s^−1^ (Supplementary Figure 2D). The optimized mass ratio of the α-Fe_2_O_3_/HPC (380 F g^−1^)/PANI/HPC (441 F g^−1^) electrode was ~0.85, according to the corresponding C_sp_ value at 50 mV s^−1^ to maintain charge balance (Figure 5A). Based on the separate potential window, the α-Fe_2_O_3_/HPC//PANI/HPC ASC can still exhibit an ideal capacitive characteristic at 2.0-V work voltage.

The CV profiles of the α-Fe_2_O_3_/HPC//PANI/HPC ASC deviate only slightly from the quasi-rectangular shape as the scan rate increases (10–200 mV s^−1^), which indicates good reversibility (Figure 5B). A couple of redox peaks in each CV curve is attributed to faradaic reactions of the electrolyte ions insertion/extraction. Nearly symmetric GCD curves were observed at 1–10 A g^−1^ (Figure 5C), indicating an excellent coulombic efficiency and superior capacitance of the α-Fe_2_O_3_/HPC//PANI/HPC ASC. The maximum C_asy_ of α-Fe_2_O_3_/HPC//PANI/HPC ASC is 212 F g^−1^ at 1 A g^−1^ (Figure 5D), superior to these published α-Fe_2_O_3_/C//MnO_2_ (Dong et al., 2018), Fe_2_O_3_/N-CNT//CuCo_2_O_4_ (Gnana Sundara Raj et al., 2020), and CF (carbon fiber)-rGO/Fe_2_O_3_//CF-MnOx (Serrapede et al., 2019), comparable to that of α-Fe_2_O_3_@C//CNTs-COOH (Xu et al., 2019) (Supplementary Table 1). Figure 5E shows the C_asy_ vs. cycle number plot of the α-Fe_2_O_3_/HPC//PANI/HPC ASC, conducted at 1 A g^−1^, which is only 5.8% decay of the original capacity after 5,000 cycles. Supplementary Figure 3 displays the Nyquist plot of the α-Fe_2_O_3_/HPC//PANI/HPC ASC at the first cycle and after the 5,000th cycle. The corresponding charge transfer resistances are 8.1 and 14 Ω, respectively. These small resistance values indicate that the as-assembled ASC has good conductivity. Moreover, Figure 5F shows the Ragone plot of the α-Fe_2_O_3_/HPC//PANI/HPC ASC suggesting a relationship of energy density (E) vs. power density (P). The as-fabricated α-Fe_2_O_3_/HPC//PANI/HPC ASC has a gravimetric E value of 117 Wh kg^−1^ at 1.0 kW kg^−1^. At the highest *P*-value of 9.3 kW kg^−1^, the E value is maintained at 102 Wh kg^−1^. The E and P values were compared with previously published devices, as listed in Supplementary Table 1, such as Fe_2_O_3_@PPy//MnO_2_ (Liang et al., 2018), porous Fe_2_O_3_/N-CNT//CuCo_2_O_4_ (Gnana Sundara Raj et al., 2020), α-Fe_2_O_3_/G//CoNi-layer double hydroxide/CNT (Chen et al., 2015), α-Fe_2_O_3_//ZnCo_2_O_4_@MnO_2_ (Ma et al., 2015), Fe_2_O_3_@PPy//MnO_2_ thin film (Le et al., 2020), and Fe_2_O_3_//MnO_2_ thin film (Gund et al., 2015).

Based on these results, the α-Fe_2_O_3_/HPC//PANI/HPC ASC exhibits superior electrochemical behaviors arising from the synergistic interaction of the components. The HPC, as a robust scaffold, performs an important function to accommodate the volume variation of α-Fe_2_O_3_ or PANI and impedes the erosion and deformation of the as-prepared electrode. The high conductivity of HPC can enhance the integrated conductivity of α-Fe_2_O_3_/HPC and provide rapid electron transmission channels, resulting in a small charge transfer resistance. Furthermore, the well-ordered α-Fe_2_O_3_ or PANI nanorod arrays were decorated in the porous surfaces of HPC to enhance the pseudocapacitance and cycle stability resulting from effective avoidance of swelling/shrinking after long-term cycling. Therefore, the α-Fe_2_O_3_/HPC//PANI/HPC ASC presents exceptional capacitor properties and is therefore an attractive candidate in commercial energy storage devices.

## Conclusions

In summary, a facile hydrothermal route was used to fabricate the α-Fe_2_O_3_/HPC electrode. Compared to α-Fe_2_O_3_ (370 F g^−1^), the enhanced C_sp_ value of the α-Fe_2_O_3_/HPC anode is 706 F g^−1^. Furthermore, assembled α-Fe_2_O_3_/HPC//PANI/HPC ASC device delivers a maximum E value of 117 Wh kg^−1^ at 1.0 kW kg^−1^ and retains 102 Wh kg^−1^ at 9.3 kW kg^−1^, accompanied with excellent capacity retention by 5.8% loss in original capacitance. This study offers a plausible way to assemble α-Fe_2_O_3_ ASCs with excellent electrochemical properties as next-generation energy storage devices.

## Data Availability Statement

The original contributions presented in the study are included in the article/Supplementary Materials, further inquiries can be directed to the corresponding author/s.

## Author Contributions

PY wrote the paper and designed the study. WD prepared the experiments. YJ polished the manuscript. All authors contributed to the article and approved the submitted version.

## Conflict of Interest

The authors declare that the research was conducted in the absence of any commercial or financial relationships that could be construed as a potential conflict of interest.
