# Supplementary material for: Porous Fe2O3 Nanorods on Hierarchical Porous Biomass Carbon as Advanced Anode for High-Energy-Density Asymmetric Supercapacitors
Source: Front Chem. 2020 Nov 26;8:611852. doi: 10.3389/fchem.2020.611852 (PMC7726331; doi:10.3389/fchem.2020.611852)
Supplement: Supplementary file 1 [file Table_1.DOCX]

Supplementary Material





Figure S1. GCD profiles of α-Fe2O3/HPC at current densities ranged from 1 to 10 A g^-1^.


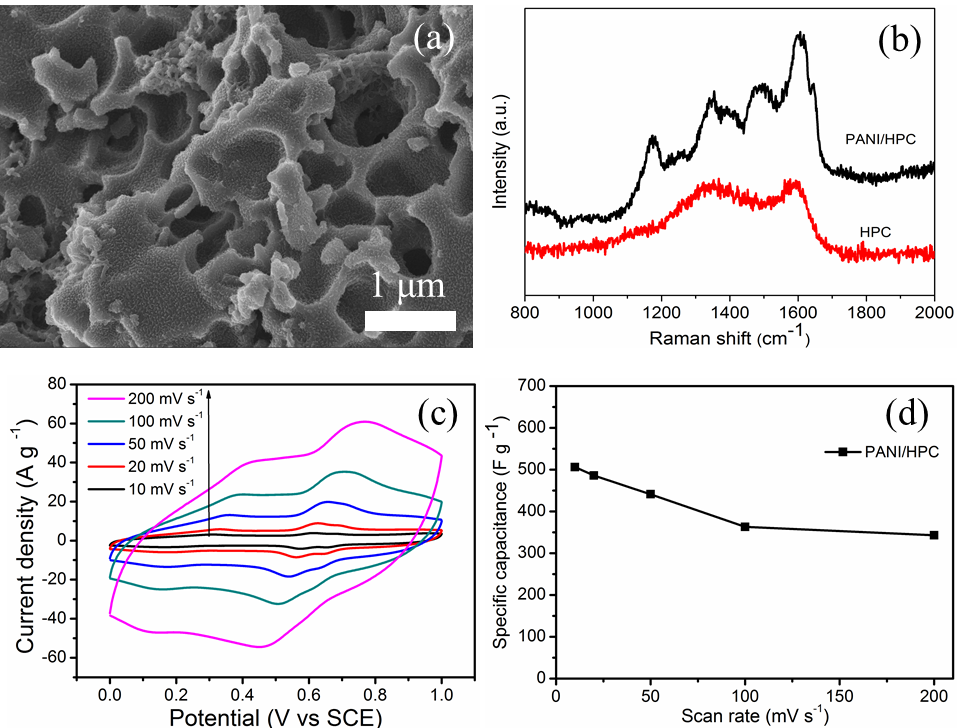


**Figure S2**. Characterization of deposited PANI nanorods arrays on HPC. (a) SEM image, (b)Raman patterns, (c) CV curves at different scan rates and (d) specific capacitance versus scan rates.





**Figure S3**. Nyquist plot of α-Fe_2_O_3_/HPC//PANI/HPC ASC before and after cycle in the frequency range of 100 kHz-0.01 Hz. The inset is the magnified view.

Table S1. Electrochemical properties of α-Fe2O3/HPC//PANI/HPC ASC and other reported ASCs in two-electrode system

| ASC devices | Casy (F g^-1^) | Cell voltage | E  (Wh kg^-1^) | P  (W kg^-1^) | Ref. |
| --- | --- | --- | --- | --- | --- |
| α-Fe_2_O_3_/HPC//PANI/HPC | 212, 1 A g^-1^ | 2 V | 117 | 1000 | This work |
| α-Fe_2_O_3_/C//MnO_2_ | 55, 1 A g^-1^ | 2 V | 30.7 | ~800 | Dong et al. 2018 |
| Fe_2_O_3_/N-CNT//CuCo_2_O_4_ | 97, 1 A g^-1^ | 1.3 V | 22.8 | 216 | [Gnana Sundara Raj et al. 2020](#_ENREF_9) |
| CF (carbon fiber)-rGO/Fe_2_O_3_//CF-MnOx | 50, 1 A g^-1^ | 1.2 V | 25 | 1000 | [Serrapede et al. 2019](#_ENREF_30) |
| α-Fe_2_O_3_@C//CNTs-COOH | 221, 1 A g^-1^ | 1.8 V | 77 | 1380 | [Xu et al. 2019](#_ENREF_35) |
| Fe_2_O_3_-P//MnO_2_ | / | / | 57.3 | 1404 | [Liang et al. 2018](#_ENREF_20) |
| α-Fe_2_O_3_/G//CoNi-layer double hydroxide/CNT | 252.4, 0.5 A g^-1^ | 1.5-1.7 V | 98 | 22800 | [Chen et al. 2015](#_ENREF_4) |
| α-Fe_2_O_3_//ZnCo_2_O_4_@MnO_2_ | 161, 2.5 mA cm^-2^ | 1.3 V | 37.8 | 648 | [Ma et al. 2015](#_ENREF_24) |
| Fe_2_O_3_@PPy//MnO_2_ thin film | 97.9, 0.5 mA cm^-2^ | 2 V | 51.2 | 285.4 | [Le et al. 2020](#_ENREF_18) |
| Fe_2_O_3_//MnO_2_ thin film | 75, 1.28 A g^-1^ | 2 V | 41.8 | 1300 | [Gund et al. 2015](#_ENREF_10) |
